# Supplementary material for: 21st Century drought-related fires counteract the decline of Amazon deforestation carbon emissions
Source: Nat Commun. 2018 Feb 13;9:536. doi: 10.1038/s41467-017-02771-y (PMC5811599; doi:10.1038/s41467-017-02771-y)
Supplement: Supplementary file 1 — Supplementary Information [file 41467_2017_2771_MOESM1_ESM.pdf]

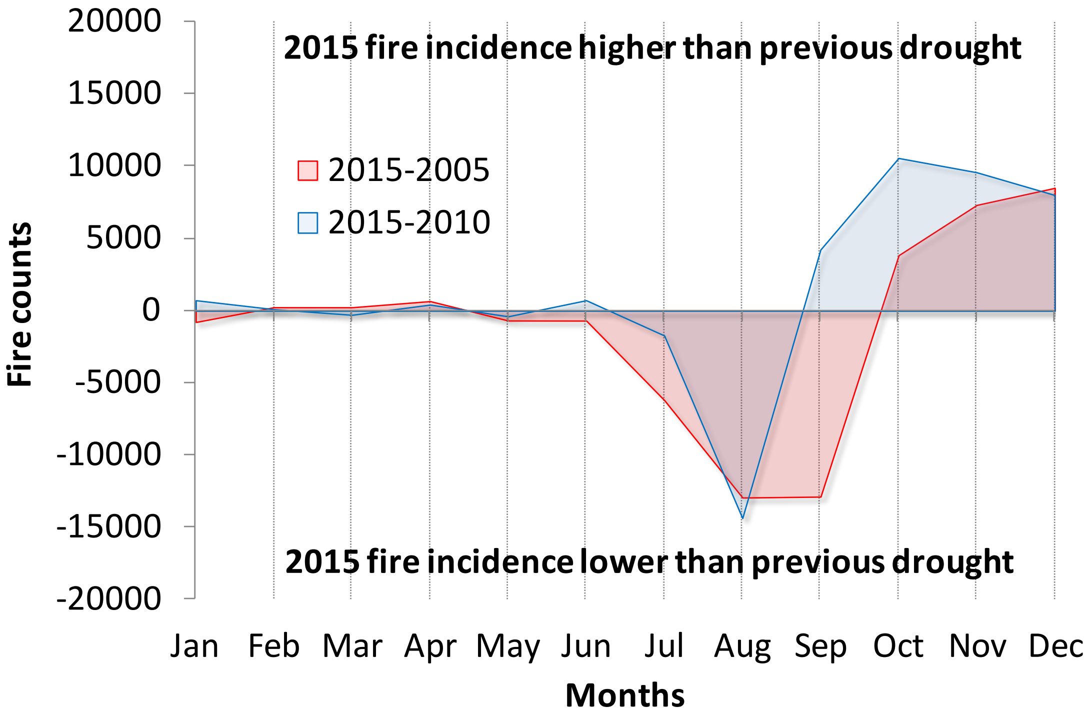

Supplementary Figure 1. Quantification of the difference between total monthly active fires detected during the 2015 drought and the 2005 and 2010 drought years. Note that negative values indicate lower fire incidence during 2015 in relation to previous droughts, while positive values indicate the opposite.

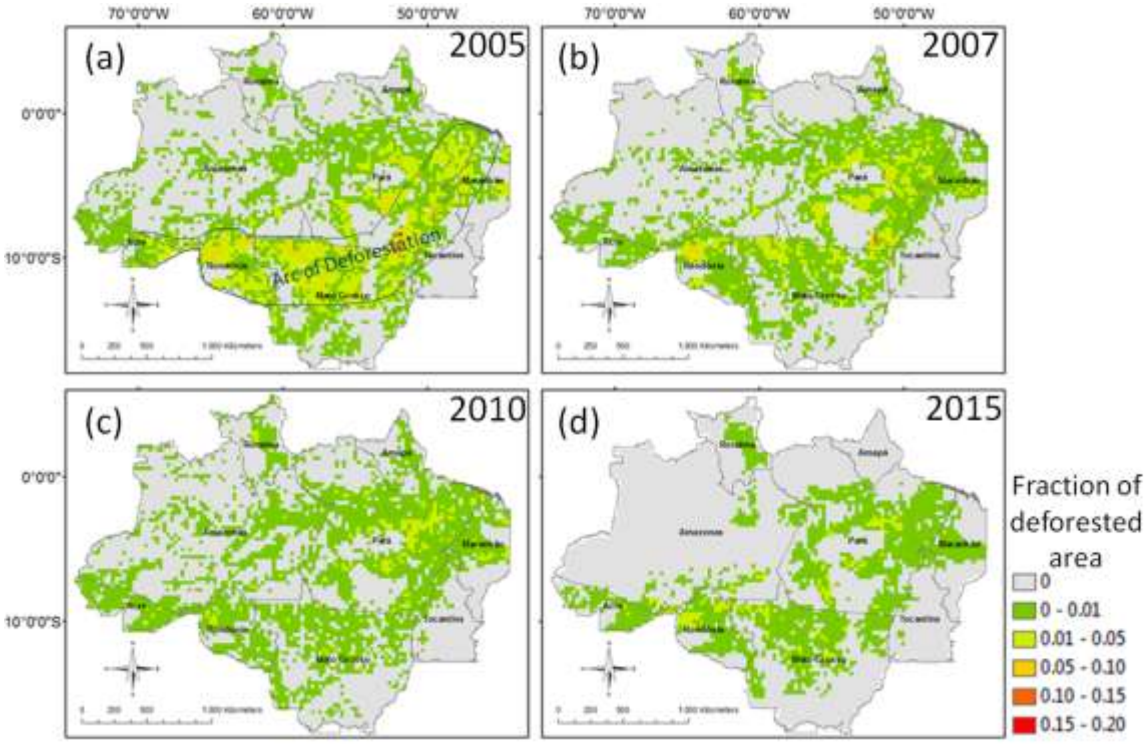

Supplementary Figure 2. Fraction deforested in each grid cell (0.25^{o} spatial resolution) for the analysed drought years (a) 2005, (b) 2007, (c) 2010 and (d) 2015. Grey background indicates no deforestation in that specific year. Dark blue polygon in (a) approximately limits the area corresponding to the arc of deforestation.

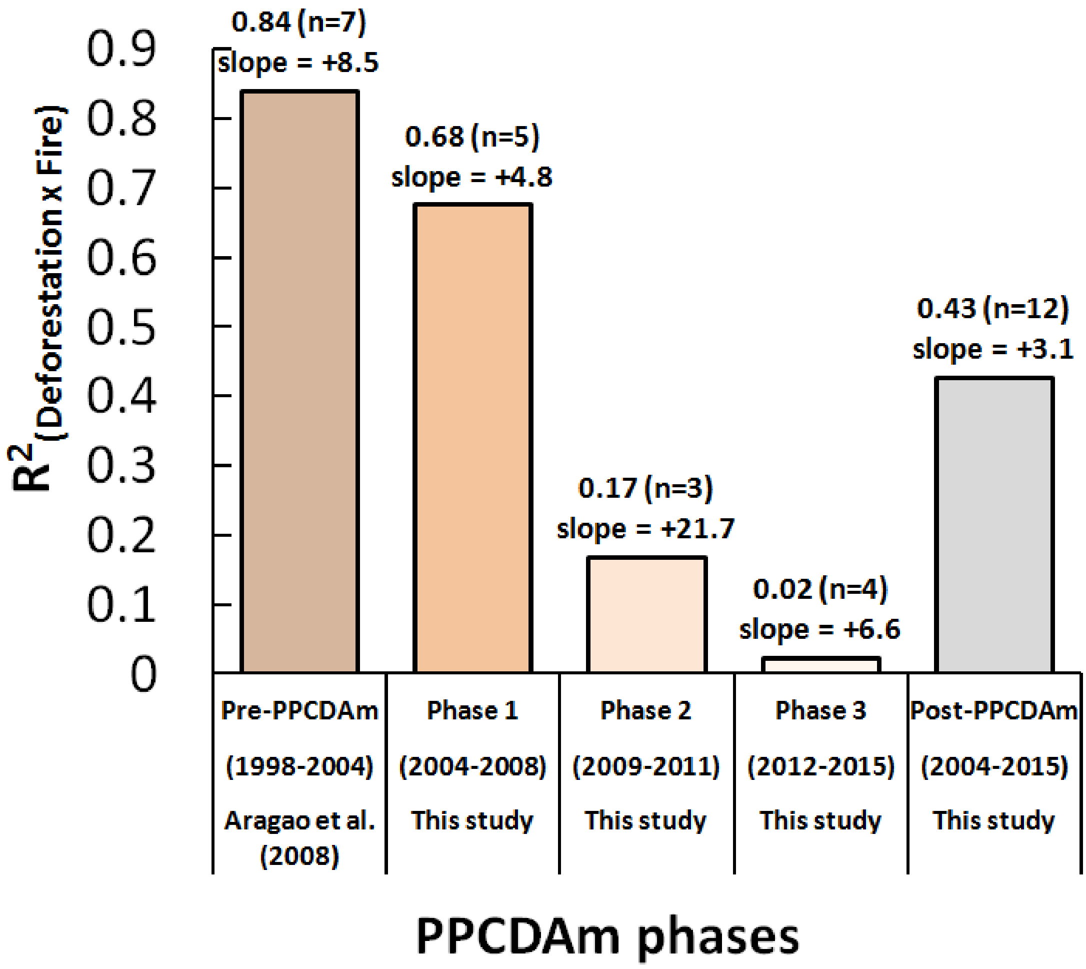

Supplementary Figure 3. Coefficient of determination for the relationship between annual deforestation rates and active fire counts. Bars depict different phases of the Action Plan for Prevention and Control of Deforestation in Amazonia (PPCDAm). In parentheses the number of years (n) used in the regression is displayed. All regression slopes are positive. Pre-PPCDAm R^2 value, n and slope are from reference 1.

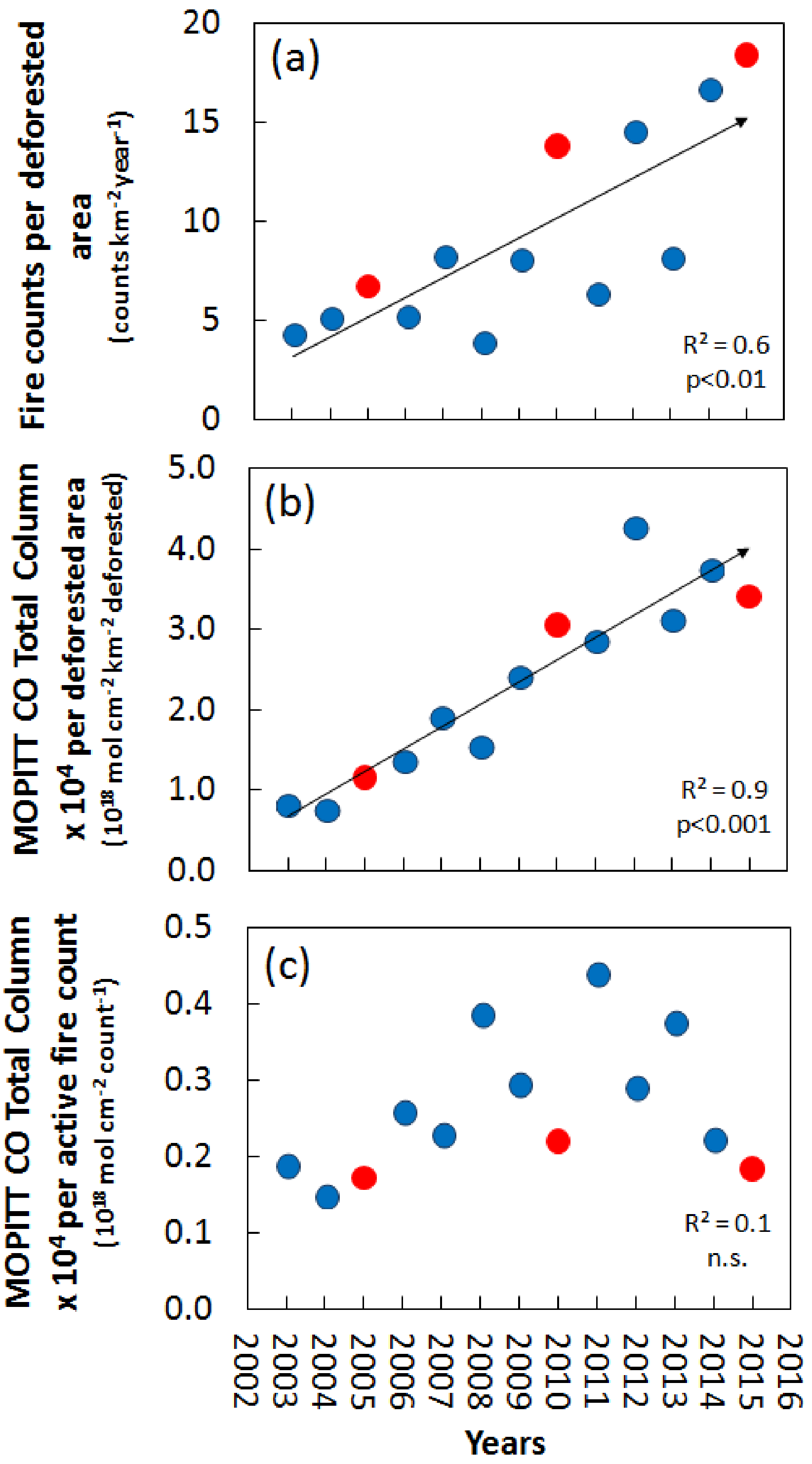

Supplementary Figure 4. Temporal trends. Linear trends (2003-2015) of annual (a) active fire counts normalised by the area deforested, (b) Measurements of Pollution in the Troposphere (MOPITT) CO total column data normalised by the area deforested and (c) MOPITT CO total column normalised by active fire counts. Linear trends (black lines) are shown for statistically significant data. In a-c R^2 is the coefficient of determination, p is the probability calculated at 95% confidence level and n.s. indicate non-significant trends.

Supplementary References

[1] Aragão, L. E. O. C. *et al*. Interactions between rainfall, deforestation and fires during recent years in the Brazilian Amazonia. *Philosophical Transactions of the Royal Society of London B: Biological Sciences*, **363**(1498), 1779-1785 (2008).
